# Role of the Unique Secreted Peptide Adropin in Various Physiological and Disease States

**DOI:** 10.3390/biom14121613

**Published:** 2024-12-17

**Authors:** Zahra Hasanpour-Segherlou, Andrew A. Butler, Eduardo Candelario-Jalil, Brian L. Hoh

**Affiliations:** 1 Department of Neurosurgery, College of Medicine, University of Florida, Gainesville, FL 32610, USA; zahra.hasanpoursegherlou@neurosurgery.ufl.edu; 2 Department of Pharmacology and Physiological Sciences, Saint Louis University, Saint Louis, MO 63104, USA; andrew.butler@health.slu.edu; 3 Department of Neuroscience, College of Medicine, University of Florida, Gainesville, FL 32610, USA; ecandelario@ufl.edu

**Keywords:** adropin, endothelial cells, metabolic syndrome, cardiovascular disease, cerebrovascular disorders, subarachnoid hemorrhage

## Abstract

Adropin, a secreted peptide hormone identified in 2008, plays a significant role in regulating energy homeostasis, glucose metabolism, and lipid metabolism. Its expression is linked to dietary macronutrient intake and is influenced by metabolic syndrome, obesity, diabetes, and cardiovascular diseases. Emerging evidence suggests that adropin might be a biomarker for various conditions, including metabolic syndrome, coronary artery disease, and hypertensive disorders complicating pregnancy. In cerebrovascular diseases, adropin demonstrates protective effects by reducing blood–brain barrier permeability, brain edema, and infarct size while improving cognitive and sensorimotor functions in ischemic stroke models. The protective effects of adropin extend to preventing endothelial damage, promoting angiogenesis, and mitigating inflammation, making it a promising therapeutic target for cardiovascular and neurodegenerative diseases. This review provides a comprehensive overview of adropin’s multifaceted roles in physiological and pathological conditions, as well as our recent work demonstrating adropin’s role in subarachnoid hemorrhage-mediated neural injury and delayed cerebral infarction.

## 1. Introduction

In 2008, Kumar et al. [1] identified adropin as a secreted factor that links dietary macronutrient intake with energy homeostasis and lipid metabolism. Adropin (derived from the Latin roots “aduro” [to set fire to] and “pinquis” [fats or oils]) is encoded by the Energy Homeostasis Associated gene (gene symbol: Enho) [1]. In lean mice, high-fat diets rapidly increased liver Enho expression, while fasting led to a decrease compared to controls. However, in cases of diet-induced obesity (DIO) or genetically induced obesity, liver Enho expression declined, linking it to metabolic disorders in obesity. For mice with DIO, both transgenic overexpression and systemic treatment with adropin reduced liver fat accumulation and insulin resistance independently of reducing food intake or adiposity. Adropin influenced the expression of lipogenic genes in white adipose tissue. Thus, adropin appears to be a critical factor in managing glucose and lipid balance, protecting against metabolic syndrome related to obesity [1].

**Figure 1:**
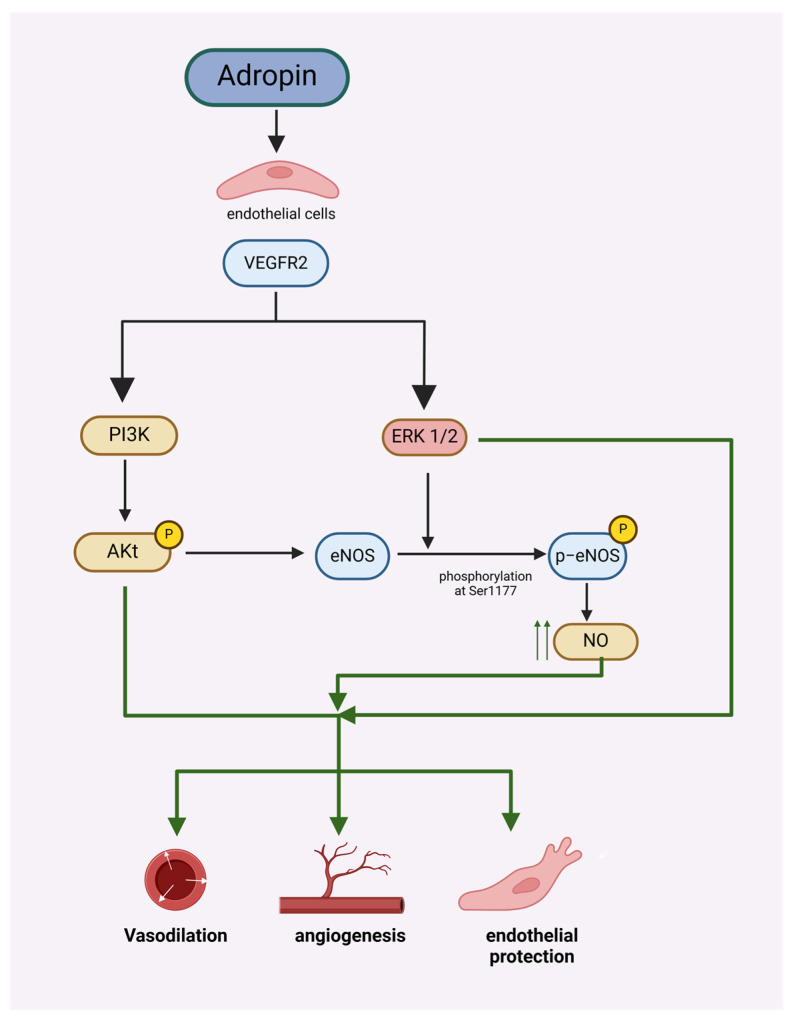
Adropin protects endothelial cells and promotes angiogenesis by stimulating the phosphorylation of eNOS at Ser1177 through the VEGFR2-phosphatidylinositol 3-kinase-Akt and VEGFR2-extracellular signal-regulated kinase 1/2 pathway.

Metabolic syndrome is a cluster of disorders that increase the risk of atherosclerotic cardiovascular disease, such as heart attacks, strokes, peripheral vascular disease, insulin resistance, and type II diabetes. It is characterized by central obesity, insulin resistance, hypertension, and atherogenic dyslipidemia [2]. Currently, cardiovascular disease is identified as a major cause of mortality around the world, and this situation is estimated to remain for many years to come, thus bringing a considerable burden to the world’s health resources [3]. Endothelial dysfunction is recommended as an independent risk factor for cardiovascular disorders [4]. Endothelial cell (EC) homeostasis is maintained, in part, through the synthesis of nitric oxide (NO) from the precursor L-arginine by the enzyme endothelial NO synthase (eNOS). Aside from exerting several critical anti-inflammatory, antithrombotic, and antiatherosclerotic roles within blood vessels, NO also promotes postnatal angiogenesis and reparative vasculogenesis. Furthermore, there is good evidence that NO bioavailability serves an important role in metabolic regulation and insulin sensitivity [5,6,7]. Adropin protects endothelial cells (human umbilical vein and coronary artery endothelial cells) by stimulating the phosphorylation of eNOS at Ser1177 through the VEGFR2-phosphatidylinositol 3-kinase-Akt and VEGFR2-extracellular signal-regulated kinase 1/2 pathways in these cells [8], and also promote angiogenesis through the activation of VEGFR2 signaling pathways (Figure 1) [9].

Adropin is widely expressed and has been detected in various tissues of rats, including the brain (vascular area, pia mater, neuroglial cell, and neurons), cerebellum (neuroglial cells, Purkinje cells, vascular areas, and granular layer), kidneys (glomerulus, peritubular interstitial cells, and peritubular capillary endothelial cells), heart (endocardium, myocardium, and epicardium), liver (sinusoidal cells), and pancreas (serous acini) [10]. In nonhuman primates, Enho expression is primarily found in the central nervous system, especially in the amygdala and hypothalamus, with lower levels in peripheral tissues such as the liver, kidney, lung, and adrenal glands [11]. Low plasma adropin levels were correlated with higher fasting glucose, plasma leptin, and apolipoprotein C-III levels, and during high-sugar diet consumption, lower adropin predicted greater weight gain and metabolic issues in nonhuman primates [11]. In humans, circulating adropin levels are correlated with indicators of metabolic homeostasis, including lipoprotein metabolism and insulin resistance [12].

Adropin gained attention not only as a potential biomarker for metabolic and cardiovascular conditions but also as a therapeutic agent due to its role in regulating energy homeostasis and endothelial function. In this review, we aim to explore the roles of adropin in metabolic syndrome, cerebrovascular and cardiovascular diseases, as well as our recent work demonstrating adropin’s role in subarachnoid hemorrhage-mediated neural injury and delayed cerebral infarction.

### 1.1. Sex Hormones and Adropin

Adropin is widely expressed, suggesting pleiotropic roles and complex regulation [1]. Given that adropin was initially studied as a “hepatokine” [1], many studies have focused on the regulation and role of adropin expressed in the liver. In a study involving C57BL/6J mice, menopause induced through ovariectomy (OVX) reduced hepatic Enho mRNA expression levels [13]. OVX is associated with visceral adiposity, hepatic steatosis, and increased risk for cardiovascular disease, which is alleviated upon adropin treatment, suggesting that adropin can improve metabolic homeostasis in the context of hypogonadism [13]. Additionally, an increase in Enho expression and elevated adropin levels in response to 17β-estradiol (E2), an estrogen steroid hormone, were observed, correlating with improved metabolic outcomes [13]. Estrogen receptor alpha (ERα) inhibits fatty acid synthesis in female mice under a high-fat diet (HFD) but not in male mice or those lacking ERα in the liver, playing a sex-specific role in regulating hepatic fat metabolism [14]. Enho expression in the liver follows a similar pattern and increases in female mice under HFD but not in male mice or those lacking the ERα receptor [15]. This study identified adropin as the hepatokine that better predicts sex-specific and hepatic ERα-dependent regulation of hepatic metabolism under different hormonal and nutritional conditions [15].

In other studies on polycystic ovary syndrome (PCOS), a relationship between sex hormones and adropin was demonstrated. PCOS, which can cause hormonal, metabolic, and reproductive abnormalities, is linked to an elevated risk of type 2 DM (T2DM) and cardiovascular disease due to atherosclerosis, metabolic syndrome, hyperlipidemia, hypertension, obesity, and oxidative stress [16]. In cases of PCOS, adropin levels are generally decreased in both serum and follicular fluid [17,18,19], though some studies have found no significant difference [20]. Notably, a meta-analysis highlighted that the decrease in adropin is more pronounced in overweight PCOS patients [21], and a meta-regression analysis suggested that factors like age, glucose ratio, and luteinizing hormone (LH) levels could contribute to the observed heterogeneity [22]. These findings underscore the importance of estrogen in modulating adropin levels and highlight its potential role in protecting against adverse metabolic conditions. However, caution is needed when interpreting these studies and other studies reporting adropin as a biomarker, as different exploratory assays were used to measure circulating adropin levels. Moreover, the relationship between circulating levels and tissue expression remains unclear.

### 1.2. Adropin and Hypertensive Disorders Complicating Pregnancy and Gestational Diabetes Mellitus

Hypertensive disorders complicating pregnancy (HDCP) are associated with decreased adropin levels [23]. Both maternal and umbilical cord adropin levels decrease in preeclamptic pregnancies compared to controls, with significantly lower levels observed in severe cases compared to mild ones. These findings suggest that adropin could potentially serve as a predictive marker for the presence and severity of preeclampsia [24,25]. Conversely, in another study, serum adropin levels increased in women with preeclampsia; this elevation may serve as a compensatory mechanism [26]. In cases of intrauterine growth retardation/restriction (IUGR), adropin levels decrease in both cord and maternal blood. However, in severe IUGR cases, maternal adropin levels increase, possibly as a regulatory mechanism against placental dysfunction [27,28]. Gestational diabetes mellitus (GDM) presents a complex relationship with adropin levels, with increases observed in both GDM 1 and GDM 2 during different trimesters, while decreases are noted in maternal and cord serum levels during specific gestational weeks [29,30,31,32,33]. Additionally, cord blood adropin levels increase in infants of GDM mothers [34]. These changes in adropin levels in different disorders during pregnancy should be adjusted based on the gestational age, as in the context of preterm delivery, cord blood adropin levels decrease and are positively correlated with gestational age and placental weight [35].

Conversely, in investigations focusing on pregnancy, a pivotal period demanding intricate cellular adaptations, the epidermal growth factor domain-specific O-linked GlcNAc transferase–adropin axis in decidual cells was found impaired, potentially linking metabolic disorders like obesity to adverse pregnancy outcomes [23].

### 1.3. Adropin and Coronary Artery Diseases

Lower serum adropin levels have been consistently associated with various cardiac conditions, serving as potential biomarkers for disease onset and severity. For instance, in patients with microvascular angina (cardiac syndrome X, CSX), serum adropin levels were significantly reduced, with adropin identified as an independent risk factor for CSX [36]. Similarly, in individuals with acute myocardial infarction (AMI), decreased serum adropin levels were linked to the presence of AMI in Coronary Artery Disease (CAD) patients, with adropin serving as an independent predictor for AMI [37], a possible role of adropin in CAD prevention [38], and the presence of good coronary collateral circulation [39]. Low adropin levels are correlated with adverse outcomes in CAD patients, such as the increased risk of long-term recurrent myocardial infarction and higher severity of CAD, as assessed by the SYNTAX score [40]. Additionally, investigations into the genetic landscape of CAD have identified polymorphisms in genes related to adropin production, further implicating adropin in the pathogenesis of CAD [41]. A study found that adropin enhanced the survival and therapeutic potential of mesenchymal stem cells (MSCs) in myocardial infarction by activating the Akt and ERK1/2 pro-survival pathways, reducing inflammation, and improving cardiac function while reducing myocardial fibrosis [42].

Studies on Coronary Artery Ectasia (CAE) found that lower serum adropin levels are associated with CAE, suggesting that adropin could be a significant predictor and independent risk factor for the condition [43,44]. Studies on heart failure (HF) have shown that plasma adropin levels increase with the severity of HF, are higher in patients with cardiac cachexia, and correlate with brain natriuretic peptide (BNP) and NYHA class, suggesting a role for adropin in the pathogenesis and severity of HF [45,46,47]. Studies on cardiomyopathies have shown that adropin regulated mitochondrial fuel substrate utilization in cardiac cells, protected against myocardial fibrosis, and improved diastolic function, suggesting its potential role in treating conditions like diabetic cardiomyopathy and doxorubicin-induced cardiomyopathy [48,49,50]. Serum adropin levels were elevated in Kawasaki disease patients, particularly those with coronary artery lesions [51].

### 1.4. Adropin’s Role in Hypertension

Adropin protects endothelial cells by stimulating the phosphorylation of eNOS in these cells [8]. Endothelial dysfunction in hypertension may play a role in triggering and advancing vascular inflammation, vascular remodeling, and atherosclerosis, and it is independently linked to a higher risk of cardiovascular events [52]. Adropin level has been shown to correlate with hypertension. Observational studies indicate that individuals with hypertension exhibit significantly lower plasma adropin levels compared to normotensive controls [40,53]. Antihypertensive medications such as amlodipine and valsartan have been demonstrated to increase adropin levels after 12 weeks of treatment [54]. However, the predictive value of adropin for target organ damage in hypertensive patients remains controversial [40]. Moreover, no studies have determined whether adropin affected blood pressure in animal models. These results are correlative and do not indicate causality.

### 1.5. Adropin’s Role in Atherosclerosis

Experimental studies highlight adropin’s ability to mitigate the progression of this cardiovascular disorder through several mechanisms. One study demonstrated that the induction of lipoprotein lipase (LPL) by the non-coding RNA HDAC11-AS1 may be mediated by adropin, leading to a reduction in atherosclerosis in mice [55]. This effect is attributed to adropin-mediated histone deacetylation, which enhances LPL expression by activating AMP-activated protein kinase (AMPK) [55]. Another study underscores adropin’s role in suppressing inflammatory responses, inhibiting foam cell formation, and reducing the migration and proliferation of vascular smooth muscle cells [56]. These actions collectively contribute to the attenuation of atherosclerotic lesion development [56]. Moreover, studies have delved into the complex interplay between lifestyle factors and vascular health, revealing that short-term adverse lifestyle changes induce vascular insulin resistance, accompanied by decreased plasma adropin levels, particularly in young, healthy men [57]. The anti-inflammatory and lipid-regulating properties of adropin suggest its potential as a therapeutic target for atherosclerosis, offering a novel approach to reducing the burden of this disease. However, adropin transgenic mice do not prevent atherosclerosis, as observed with low-density lipoprotein receptor (LDLR) deficiency [58]. The results of those studies suggest that cholesterol and intermediates in cholesterol metabolism inhibit adropin/Enho expression. However, overexpression did not affect cholesterol levels.

### 1.6. Adropin’s Role in Obesity

The link between obesity and glucose and lipid metabolism disturbances is well established [59]. Peptide hormones play a key role in regulating overall energy balance in the body, and adropin is also associated with this process [1,59]. In children, higher plasma adropin levels have been associated with lower waist-to-hip ratios, lower insulin resistance, and lower body fat percentage in both girls and boys [60]. However, no significant differences were observed between genders or in lipid profiles among high versus low adropin subjects, suggesting a complex interaction with body composition [59,60,61,62,63]. Similarly, observational studies highlight a decrease in adropin levels with obesity and aging, with significant differences observed between prepubertal and pubertal children and varying associations with obesity depending on developmental stage [64], with higher concentrations found in males [65].

In adolescents, lower adropin levels have been associated with high-density lipoprotein cholesterol (HDL-C) levels and have been shown to increase with exercise [66]. Bariatric surgery has been observed to increase circulating adropin levels, peaking 3 months after surgery [65], which may contribute to the long-term metabolic improvements seen post-surgery through enhanced secretion of anti-inflammatory and insulin-sensitizing factors [67,68]. In obese children with metabolic syndrome, lower levels of adropin were noted, and adropin was identified as a protective factor against metabolic syndrome, indicating its potential as a biomarker for this condition [69]. Interestingly, in conditions such as Prader–Willi syndrome (PWS), characterized by severe obesity, adropin levels were found to be higher compared to controls [70]. White adipose tissue regulates lipid and carbohydrate metabolism by storing triacylglycerol and releasing various hormones and metabolic factors that interact with peripheral tissues and the brain [71,72]. In adipocytes, the expression of Gpr19 and Enho genes follows a similar trend, suggesting that Gpr19 could be a potential receptor for adropin [73]. However, no studies have directly confirmed this relationship. Collectively, these findings suggest that adropin may serve as a biomarker for obesity and related metabolic syndromes, with potential implications for therapeutic interventions targeting metabolic dysregulation. Worth mentioning is the fact that the studies cited used different assays to measure adropin levels. While some assays, such as ELISA, are widely validated and commonly used in research, others may require more cautious interpretation. This variability in assay choice could influence the comparability of the results, and readers should consider these differences when drawing conclusions across studies.

### 1.7. Adropin’s Role in Obstructive Sleep Apnea (OSA)

Obstructive sleep apnea (OSA) is a prevalent sleep-related breathing disorder resulting from partial or complete upper airway obstruction [74]. OSA has been independently linked to endothelial dysfunction, inflammation, hypertension, dysregulation of the hypothalamic–pituitary–adrenal axis, increased arterial stiffness, impaired glucose metabolism, and cognitive and psychomotor deficits [74].

Severe OSA in adults is associated with markedly lower adropin levels compared to moderate OSA, healthy controls [74,75], and obese males without OSA [76]. In OSA, decreased adropin levels suggest its utility as an early biomarker for predicting endothelial dysfunction prior to the manifestation of clinical symptoms. However, serum NO levels do not significantly differ between OSA patients and healthy controls [77]. In pediatric OSA patients, adropin levels are significantly reduced but return to normal values following adenotonsillectomy [78,79]. These findings underscore the complex relationship between adropin and OSA, emphasizing its potential role in both the diagnosis and management of this condition.

### 1.8. Adropin’s Role in DM

Research using different animal models, spanning multiple studies, sheds light on adropin’s multifaceted role, particularly focusing on its molecular mechanisms within the context of diabetes mellitus (DM) [80,81,82,83,84,85,86,87]. Studies have shown adropin’s involvement in glucose and lipid metabolism regulation [80,81]. For instance, adropin treatment has been shown to enhance insulin sensitivity, ameliorate insulin resistance, and promote glucose utilization while suppressing fatty acid oxidation, thus favoring a metabolic shift toward carbohydrate metabolism [80,82,88]. Mechanistically, adropin treatment activates key molecules in insulin signaling pathways, such as Akt and GLUT4, sensitizing tissues to insulin action [81]. Additionally, adropin affects glucose homeostasis by modulating enzymes and transcription factors involved in glucose and lipid metabolism, such as pyruvate dehydrogenase and PPARα [81]. Furthermore, adropin has been found to regulate hepatic glucose production by inhibiting gluconeogenic enzymes and the modulation of AMPK signaling [88].

Observational studies in clinical settings have revealed elevated serum adropin levels in T2DM patients compared to healthy controls, suggesting a potential association with disease pathogenesis [89]. However, interventional studies have provided contrasting insights, demonstrating that treatment with sitagliptin, a common antidiabetic medication, could significantly increase adropin levels in newly diagnosed T2DM patients, possibly contributing to improved glucose metabolism [90]. Adropin also plays a crucial role in metabolic dysfunction-associated fatty liver disease (MAFLD) in patients with T2D, as highlighted in a study examining the impact of liraglutide treatment. Results revealed that liraglutide not only increased serum adropin levels but also correlated with a significant reduction in liver fat content [91]. Training, particularly high-intensity interval training, has been shown to significantly elevate adropin levels in patients with T2DM, potentially contributing to improved insulin sensitivity and endothelial function [92,93]. Cross-sectional investigations have further explored the relationship between dietary factors and metabolic health in T2DM, highlighting the potential impact of dietary insulin index on metabolic profiles, albeit without significant associations with adropin levels [94].

### 1.9. Endothelial Dysfunction in T2DM

Adropin plays a significant role in T2DM and associated cardiovascular disorders by influencing endothelial function and atherosclerosis, independently associated with angiographic severity of coronary atherosclerosis, suggesting that serum adropin serves as a novel predictor of coronary atherosclerosis [95]. Studies have shown that lower plasma adropin levels are associated with endothelial dysfunction in T2DM patients, indicating its potential as a biomarker for vascular health [95,96,97]. Specifically, T2DM patients with lower adropin levels exhibited worse endothelial function and higher hemoglobin A1C levels, suggesting an independent risk factor for vascular complications [97]. Additionally, higher serum adropin levels were correlated with a reduced risk of carotid atherosclerosis and a decrease in carotid intimal–medial thickness, underscoring its protective role against atherosclerotic cardiovascular disease in T2DM patients [98]. Adropin levels also showed potential as a risk indicator for ischemic heart disease in T2DM, with significantly lower levels observed in patients with ischemic heart disease compared to those without [99]. Furthermore, although adropin levels did not significantly vary with glycemic control in chronic heart failure patients, they were associated with favorable hemodynamic changes during treatment with SGLT2 inhibitors, indicating a beneficial role in cardiac remodeling and function [100,101].

### 1.10. DM Nephropathy and Retinopathy

Serum adropin concentrations exhibit a consistent negative correlation with renal function, particularly in patients with T2DM. Notably, lower adropin levels are associated with an increased risk of developing both T2DM and diabetic nephropathy, suggesting its pivotal role in the disease progression [102,103,104]. Moreover, adropin’s role extends beyond mere association, as experimental studies demonstrate its therapeutic potential [105]. Adropin, encapsulated in ROS-responsive nanocapsules, showcases promising effects in improving renal function, mitigating oxidative stress, and regulating lipid metabolism in models of diabetic kidney disease [105]. Furthermore, clinical observations underscore adropin’s prognostic value, with low levels serving as an independent predictor of chronic kidney disease stages 1–3 in T2DM patients with chronic heart failure [106].

Recent studies have established a negative correlation between adropin levels and the severity of diabetic retinopathy (DR), with lower adropin concentrations associated with increased risk and severity of DR [107,108].

### 1.11. Adropin and Hepatic Diseases

Since adropin was originally investigated as a “hepatokine” [1], numerous studies have concentrated on the regulation and function of adropin in the liver. Adropin has been shown to correlate with dyslipidemia and related metabolic disorders. Observational human studies have revealed that plasma adropin levels were inversely related to atherogenic LDL-C levels, suggesting a potential regulatory role in hepatic lipid metabolism [58]. Research on mice indicates that the interaction between adropin and cholesterol metabolism is mostly one-way, with cholesterol and seven-oxygenated sterols primarily inhibiting adropin expression in cultured cells [109]. The concentration of angiopoietin-like protein 3, a promising target for managing cardiovascular disease, which inhibits lipid clearance, is inversely related to adropin levels in fructose-induced dyslipidemia in rhesus macaques [110]. Therefore, this study implicates the role of adropin in the complex regulatory network of insulin resistance and hypertriglyceridemia [110]. In obese adolescents with non-alcoholic fatty liver disease (NAFLD), adropin levels are significantly decreased compared to both healthy controls and patients without fatty liver disease [111]. Additionally, adropin levels are inversely correlated with oxidative stress and histological severity in NAFLD, suggesting a potential role as a predictor of coronary artery disease [112,113]. Conversely, in alcoholic liver cirrhosis, adropin concentrations are increased, and this elevation correlates with disease severity, as indicated by the Child–Pugh score [114]. Moreover, in cirrhotic patients, higher adropin levels are associated with shorter survival within 180 days, and combining serum adropin with Child–Pugh and MELD–Na scores enhances mortality prediction [115]. Furthermore, in chronic hepatitis B or C, adropin levels increase with disease progression, offering insights into fibrosis staging and monitoring [116]. However, in MAFLD, adropin levels are decreased, particularly in patients with T2DM, highlighting its potential as a marker for metabolic dysfunction [117]. In preclinical models, adropin demonstrates a protective role in diet-induced nonalcoholic steatohepatitis (NASH) by activating Nrf2 signaling and inhibiting NLRP3 inflammasome activation, suggesting its therapeutic potential in NASH prevention and treatment [118,119]. Notably, adropin regulation in the liver appears to be sex-specific and dependent on estrogen receptor alpha (ERα) in mice on an HFD [15]. These findings underscore the multifaceted involvement of adropin in liver pathophysiology and its potential as a diagnostic and therapeutic target in liver diseases.

### 1.12. Adropin and Renal Diseases

Chronic kidney disease (CKD) is linked to an age-related decline in kidney function, which is further exacerbated by conditions such as hypertension, diabetes, obesity, and primary renal disorders [120]. Cardiovascular disease is the leading cause of illness and death, with CKD recognized as both a risk factor for cardiovascular events and a condition that accelerates cardiovascular disease risk [120]. Adropin has been increasingly studied for its role in renal diseases. In a rat model of adenine-induced chronic kidney disease (CKD), adropin was found to reduce urine protein levels and 24-hour urine volume and downregulate inflammatory markers such as TIMP-1, IL-33, and MMP-2, suggesting a protective effect against renal damage and inflammation [121]. In patients undergoing hemodialysis (HD), lower serum adropin levels were significantly associated with malnutrition, inflammation, and longer HD duration, highlighting its potential involvement in the pathophysiology of CKD and its complications [122]. Studies also showed that genetic polymorphisms affecting adropin levels were associated with dyslipidemia and cardiovascular risks, further emphasizing the hormone’s role in metabolic and cardiovascular health in kidney disease patients [123,124,125]. Additionally, adropin levels were found to be inversely correlated with body composition parameters in end-stage renal disease patients, suggesting its utility as a marker for nutritional status [126]. Overall, these findings indicate that adropin plays a significant role in modulating inflammatory responses, renal function, and metabolic health in various stages of kidney disorders [127,128]. Adropin could also positively serve as a marker for metabolic health and recovery in kidney transplant patients [119,129].

### 1.13. Nutrition and Exercise Effects on Adropin

Plasma adropin concentrations strongly correlate with the intake of saturated fat [130]. Levels of adropin were found to be higher in cheese whey compared to those in the corresponding milk peptides and plasma of dairy cows [131]. Consumption of fructose, probiotic yogurt, a low-calorie diet, dietary fats, legumes, and nuts increases adropin levels, while carbohydrate-rich diets decrease them [130,132,133,134,135].

Adropin promotes lipolysis, suppresses lipogenesis, and modulates adipokine expression in white rodent adipocytes, suggesting its pivotal role in regulating metabolic processes within adipose tissue [136]. Adropin appears to exert significant effects on skeletal muscle metabolism and function, as evidenced by several studies. Research indicates that adropin promotes carbohydrate oxidation over fat oxidation in muscle tissue, suggesting a role in regulating substrate preferences [137]. Additionally, alterations in adropin levels, such as downregulation observed in response to dietary changes, may impact gene expression related to lipid metabolism and oxidative stress in skeletal muscle [138]. Exercise interventions, particularly aerobic training, have been associated with increases in adropin levels, correlating with improvements in arterial stiffness and adiposity in obese adults [139]. Furthermore, various modes of exercise, including descending stair walking and elastic band resistance training, have demonstrated significant elevations in adropin levels, suggesting a potential role in enhancing vascular function and cardiometabolic health [140,141].

### 1.14. Adropin and Neurological Disorders

High levels of adropin peptide have been detected in the mouse brain [142], and Enho mRNA is abundant in both human and nonhuman primate brains [143], suggesting its relevance in regulating brain functions. Studies in nonhuman primates indicate that adropin expression follows a circadian rhythm, with transcriptional activation by RORα/γ playing a role in this gene’s transcription [58]. This could indicate a role for adropin in mediating circadian processes in the nervous system [58]. Disruptions in circadian regulation, potentially involving adropin, have been linked to neurological disorders [144,145]. In depression, decreased adropin levels have been observed, though depression subtypes do not show differences in these levels [146]. Similarly, reduced adropin levels are noted in individuals with bipolar disorders [147]. Experimental studies on Wistar albino rats indicate that adropin has a beneficial antiepileptic effect during penicillin-induced epileptiform activity [148]. In multiple sclerosis, patients exhibit decreased adropin levels, but these levels do not correlate with pituitary diameters [149,150,151]. In experimental Wistar rats subjected to chronic stress, Enho gene expression increased with chronic stress [152]. Adropin exerts its physiological effects through direct postsynaptic actions on neurons in the paraventricular nucleus, implicating it as a potential modulator of fluid balance, energy homeostasis, and cardiovascular regulation [153]. Adropin levels are elevated in acute hypoxia, leading to reduced water intake by modulating the TRPV4-CamKK-AMPK signaling pathway in the SFO. These findings suggest that adropin is a key mediator of the Water intake reduction (anti-dipsogenic) effects observed under hypoxic conditions [154]. Adropin may serve as a novel local stimulator for growth hormone gene expression in tilapia pituitary [155].

In a study using in silico expression profiling in a nonhuman primate diurnal transcriptome atlas, it was found that Enho expression followed a dynamic, diurnal pattern and was abundant in brain regions regulating appetite and autonomic function, with lower levels in the liver, lung, kidney, ileum, and some endocrine glands. Hierarchical clustering identified 426 genes co-regulated with Enho, enriched for epigenetic silencing and neural functions [11]. In a study, adropin knockout mice exhibited decreased locomotor activity and impaired motor coordination coupled with defective synapse formation, a phenotype similar to NB-3 (contactin-6) knockout mice [142]. However, no studies have directly demonstrated that adropin acted through NB-3.

### 1.15. Adropin and Neurodegenerative Diseases

Adropin has been shown to play a significant role in cognitive function across various models of metabolic and neurological diseases. In LDLR-deficient (low-density lipoprotein receptor) mice with diet-induced obesity, adropin overexpression improved cognitive performance by attenuating the negative effects of metabolic dysregulation on neuronal signaling pathways [156]. In rats, adropin administration enhanced spatial memory performance through the activation of the Akt/CREB/BDNF signaling pathway, demonstrating its potential to improve learning and memory [157]. Human studies revealed that higher adropin levels are associated with better cognitive outcomes and reduced risk of cognitive decline in older adults, suggesting a protective role against age-related cognitive impairment [143,158,159]. Furthermore, adropin’s positive correlations with mitochondrial processes and synapse function, particularly in individuals without dementia, indicate its involvement in maintaining neural health [143].

Potent neurotrophic responses in primary cultured neurons are consistent with adropin, supporting the development and function of neural networks. Increasing adropin expression using transgenesis improved spatial learning and memory, novel object recognition, resilience to exposure to new environments, and reduced mRNA markers of inflammation in old mice. Treatment with synthetic adropin peptide also reversed age-related declines in cognitive functions and affected the expression of genes involved in morphogenesis and cellular metabolism [143].

Adropin potentially plays a role in neurodegenerative conditions such as Parkinson’s disease (PD) and Alzheimer’s disease (AD) [160]. PD ranks as the second most prevalent neurodegenerative disorder and is often linked with gastric ulcers [160]. In an experimental model combining indomethacin-induced gastric ulcers with rotenone-induced PD [160], adropin effectively restored dopamine levels in the striatum and ameliorated rotenone-induced motor impairments. Additionally, it demonstrated significant gastroprotective effects, likely mediated by its antioxidant properties, evidenced by a reduction in malondialdehyde levels and increased activities in superoxide dismutase, catalase, and serum ferric reducing antioxidant power (assessing the serum’s ability to neutralize oxidative stress by measuring its capacity to reduce ferric iron to ferrous iron). Adropin reinstated the delicate equilibrium between the compromised pro-survival PI3K/Akt/murine double minute 2 pathways and the apoptotic P53/Puma pathways. Thus, adropin emerges as a promising therapeutic target for PD and its associated gastric ulcers [160]. In a separate clinical investigation [161], plasma levels of adropin were found to be diminished in the AD group and reduced in the MOTS-c (mitochondrial open reading frame of the 12S rRNA-c), acute ischemic stroke, and AD groups compared to the control. Similarly, comparable values were observed in the MS group relative to its control. Correlation analyses revealed platelet and cholesterol levels to be negatively associated with adropin levels, while platelet, lymphocyte, and triglyceride levels exhibited positive correlations with MOTS-c. This study provides novel insights, indicating that adropin may serve as a potentially significant marker in AD and MOTS-C in acute ischemic stroke and AD contexts [161].

### 1.16. Adropin and Ischemic Stroke

Adropin has demonstrated significant protective effects on the BBB in various models of neurological disorders. In ischemic conditions, adropin treatment reduced endothelial permeability by inhibiting the ROCK-MLC2 (Rho-associated kinase-myosin light chain 2)-signaling pathway, even though it did not protect junction proteins or reduce VEGF levels [162]. In aged mice subjected to ischemic stroke, adropin treatment decreased infarct volume and brain edema and improved sensorimotor and cognitive functions by reducing Matrix metalloproteinase-9 (MMP-9) activity and preserving tight junction proteins [163]. Further studies indicated that adropin reduced BBB damage by decreasing oxidative stress and neutrophil infiltration, with its protective effects dependent on eNOS activation [164]. Adropin serum levels are associated with poor clinical outcomes and greater infarcted areas in acute ischemic stroke patients [165]. Aging reduces adropin levels in the brain, which correlates with reduced eNOS and increased oxidative stress associated with age-related endothelial dysfunction and the development of aging-associated cerebrovascular dysfunction in Sprague-Dawley naïve rats [166]. In aged mice subjected to ischemic stroke, adropin markedly decreased infarct volume and brain edema and improved both sensorimotor and cognitive functions, with effects linked to reduced MMP-9 activity and preservation of tight junction proteins [163].

### 1.17. Adropin and Subarachnoid Hemorrhage

Our group has investigated the role of adropin in subarachnoid hemorrhage-mediated neural injury and delayed cerebral infarction. Current understanding of the pathophysiology of post-SAH cerebral infarction points to injury cascades involving decreased nitric oxide (NO) bioavailability and oxidative stress [167,168,169,170,171,172]. Under normal conditions, NO signaling pathways regulate cerebral blood flow (CBF) by mediating cerebral vasodilation and inhibiting platelet adhesion. However, with SAH, red blood cells lyse and release hemoglobin, which is spasmogenic. Hemoglobin scavenges NO, stimulates the production of a nitric oxide synthase (NOS) inhibitor (ADMA), and generates reactive oxygen species (ROS) and nitrogen species (RNS) [167].

**Figure 2:**
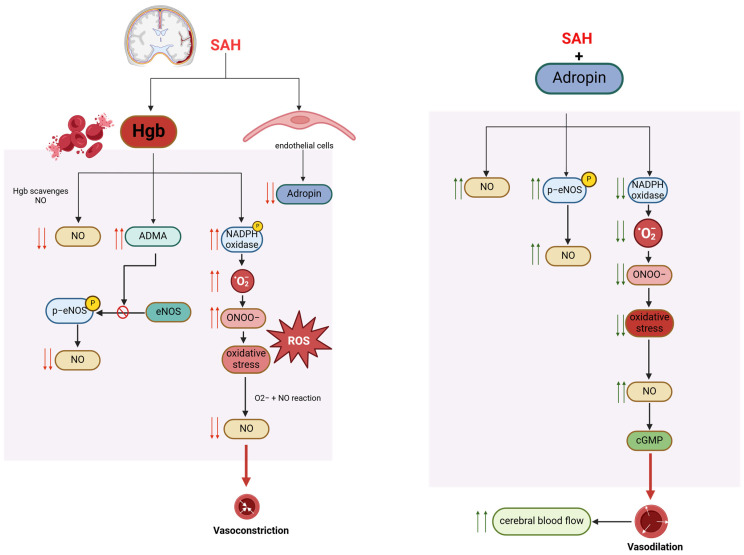
Proposed mechanisms of adropin-mediated protection in post-SAH cerebral infarction.

We showed that in endothelial cells exposed to cell-free hemoglobin as a model of subarachnoid hemorrhage, adropin prevented increased permeability and reduced macrophage migration across the endothelial monolayer [173]. In our SAH mouse model, we showed that adropin was effective against subarachnoid hemorrhage-mediated neural injury and delayed cerebral infarction through eNOS-dependent mechanisms [174]. We demonstrated that synthetic adropin treatment reduced cerebral edema, preserved tight junction proteins, and prevented microthrombosis shortly after SAH [174]. Furthermore, adropin prevents cerebral vasospasm, decreases neuronal apoptosis, and improves sensorimotor function up to seven days post-SAH, even with a 24-hour delay in administration [174]. Moreover, in intracerebral hemorrhage (ICH) models, adropin reduced brain water content and improved neurological outcomes by preserving BBB integrity [175]. Taken together, we believe that adropin has a role in protecting against subarachnoid hemorrhage-mediated neural injury and delayed cerebral infarction via a nitric oxide pathway and reduction in oxidative stress (Figure 2).

## 2. Conclusions

Adropin has emerged as a crucial regulator of metabolic, cardiovascular, and cerebrovascular physiology, demonstrating protective effects across a wide range of conditions, including metabolic syndrome, diabetes, cardiovascular diseases, and stroke. Its ability to influence energy homeostasis, lipid metabolism, and endothelial function underscores its potential as both a biomarker and a therapeutic target. In the context of cerebrovascular diseases, adropin reduces blood–brain barrier damage, brain edema, and oxidative stress while preserving cognitive and sensorimotor functions after ischemic stroke. Its therapeutic benefits extend to models of subarachnoid hemorrhage, where it decreases neuronal apoptosis and improves recovery. The inverse relationship between adropin levels and disease severity in conditions such as coronary artery disease, hypertension, and neurodegenerative diseases highlights its diagnostic significance. Future research should focus on elucidating the molecular mechanisms underlying adropin’s effects, as well as its therapeutic potential in preventing and treating cardiometabolic, cerebrovascular, and neurological disorders.

## Data Availability

Not applicable.
